# Herpes Simplex Virus type 1 DNA associated with brain region specific Tau levels measured by PET imaging

**DOI:** 10.1002/alz70862_110049

**Published:** 2025-12-23

**Authors:** Caitlin Newman, Marlene Tejeda, John J. Farrell, Congcong Zhu, Lee Wetzler, Kathryn L. Lunetta, William S Bush, Eden R. Martin, Li‐San Wang, Gerald D. Schellenberg, Margaret Pericak‐Vance, Jonathan L Haines, Lindsay A. Farrer, Richard Sherva

**Affiliations:** ^1^ Boston University, Boston, MA USA; ^2^ Boston University School of Medicine, Boston, MA USA; ^3^ Biomedical Genetics, Department of Medicine, Boston University Medical School, Boston, MA USA; ^4^ Department of Biostatistics, Boston University School of Public Health, Boston, MA USA; ^5^ Department of Population and Quantitative Health Sciences, Institute for Computational Biology, Case Western Reserve University, Cleveland, OH USA; ^6^ John P. Hussman Institute for Human Genomics, University of Miami Miller School of Medicine, Miami, FL USA; ^7^ Penn Neurodegeneration Genomics Center, Department of Pathology and Laboratory Medicine, University of Pennsylvania Perelman School of Medicine, Philadelphia, PA USA; ^8^ Penn Neurodegeneration Genomics Center, Perelman School of Medicine, University of Pennsylvania, Philadelphia, PA USA; ^9^ The John P. Hussman Institute for Human Genomics, University of Miami, Miami, FL USA; ^10^ Department of Population & Quantitative Health Sciences, School of Medicine, Case Western Reserve University, Cleveland, OH USA

## Abstract

**Background:**

Evidence suggests Herpes simplex virus type 1 (HSV‐1) infection is associated with Alzheimer's Disease (AD). Latent herpes infections may contribute to the development of amyloid and tau pathology, however, the specific relationship between HSV‐1 presence in cells and the deposition of amyloid or tau remains poorly understood.

**Method:**

HSV‐1 DNA was quantified in whole‐genome sequencing (WGS) data generated by the Alzheimer’s Disease Sequencing Project (ADSP) that were obtained from participants (**67 cases** and 505 cognitively normal controls) of ADNI and the Wisconsin Registry for Alzheimer's Prevention, of which the majority were of European ancestry. Amyloid‐β and tau PET imaging data were obtained from the ADSP phenotype harmonization consortium. The association between HSV‐1 DNA quantity and log‐transformed standardized uptake value ratios (SUVRs) of amyloid‐β and tau that were measured in several brain regions was evaluated using linear regression models including covariates for AD status, age, and gender.

**Result:**

300 participants had detectable HSV‐1 DNA. No significant associations were identified between HSV‐1 DNA and amyloid PET imaging metrics. However, we identified an association of HSV‐1 with decreased tau in numerous brain regions, but saw no association with increased tau levels once all covariates were accounted for. HSV‐1 was associated with decreased tau levels in the right hippocampus (β = ‐0.063, *p* = 7.93 × 10⁻¹¹) and left hippocampus (β = ‐0.054, *p* = 1.12 × 10⁻⁷). Additionally, we observed negative associations between HSV‐1 DNA and tau in cerebral white matter (β = ‐0.0406, *p* = 3.94 × 10⁻⁹), amygdala (β = ‐0.0547, *p* = 2.70 × 10⁻⁸), CSF (β = ‐0.0302, *p* = 7.61 × 10⁻⁸), and caudate (β = ‐0.0631, *p* = 6.81 × 10⁻¹⁵).

**Conclusion:**

Our findings suggest that the effect of HSV‐1 infection is complex and potentially protective against tau buildup in many brain regions. Conversely, tau may have a protective effect against HSV‐1. These findings highlight the need for further research to elucidate the mechanisms underlying the relationship between HSV‐1 and tau and their potential implications for neurodegenerative processes.

